# Lactuside B decreases aquaporin-4 and caspase-3 mRNA expression in the hippocampus and striatum following cerebral ischaemia-reperfusion injury in rats

**DOI:** 10.3892/etm.2013.1460

**Published:** 2013-12-24

**Authors:** PING-FA LI, HE-QIN ZHAN, SHENG-YING LI, RUI-LI LIU, FU-LIN YAN, TAI-ZHEN CUI, YU-PING YANG, PENG LI, XIN-YAO WANG

**Affiliations:** 1 Department of Laboratory Medicine, Xinxiang Medical University, Xinxiang, Henan 453003, P.R. China; 2 College of Pharmacy, Xinxiang Medical University, Xinxiang, Henan 453003, P.R. China; 3 Student Union, The Affiliated Middle School of Henan Normal University, Xinxiang, Henan 453002, P.R. China

**Keywords:** lactuside B, cerebral I/R injury, brain edema, AQP4, caspase-3, apoptosis, hippocampus, striatum

## Abstract

This study aimed to investigate the effects of lactuside B (LB) on aquaporin-4 (AQP4) and caspase-3 mRNA expression in the hippocampus and the striatum following cerebral ischaemia-reperfusion (I/R) injury in rats. Cerebral I/R injury was established in Sprague-Dawley rats by occluding the middle cerebral artery for 2 h and then inducing reperfusion. Rats in the I/R + LB groups were treated with various doses of LB following reperfusion. Neurological deficit scores and brain water content were obtained to determine the pharmacodynamics of LB. Reverse transcription polymerase chain reaction was performed to determine the expression levels of AQP4 and caspase-3 mRNA in the hippocampus and the striatum. The results of the present study indicate that LB decreased the neurological deficit scores and the brain water content. In the hippocampus, AQP4 and caspase-3 mRNA expression levels were significantly downregulated in the I/R + LB groups at 24 and 72 h following drug administration, compared with those in the I/R group (P<0.05). In the striatum, LB was also shown to significantly reduce AQP4 and caspase-3 mRNA expression levels at 24 and 72 h following drug administration, compared with those in the I/R group (P<0.05). The effects became stronger as the LB dose was increased. The most significant reductions in AQP4 and caspase-3 mRNA expression were noted in the I/R + LB 25 mg/kg and I/R + LB 50 mg/kg groups at 72 h following drug administration. The results of the present study show that LB is capable of significantly downregulating AQP4 and caspase-3 mRNA expression in the hippocampus and striatum following cerebral I/R injury in rats. The mechanism by which LB improved ischaemic brain injury may be associated with changes in AQP4 and caspase-3 mRNA expression in the hippocampus and the striatum.

## Introduction

Cerebral ischaemia is caused by a number of factors, including advanced age, hypertension, previous stroke or transient ischaemic attack and cardiac arrest (1). It predominantly results in neuronal apoptosis or neuronal death in the brain regions that are most intrinsically vulnerable, including the CA1 region of the hippocampus and the striatum (2,3). The extent of brain damage is determined by the severity of the primary injury and the intensity of the secondary injury cascades that contribute to delayed cellular destruction (4).

Following the onset of ischaemia, oxygen and glucose supply is interrupted and this causes cell mortality cascades, resulting in the breakdown of the blood-brain barrier and cerebral oedema (5). Brain oedema is one of the most common causes of disability and mortality in patients suffering from ischaemia (6). Reperfusion that occurs following focal cerebral ischaemia exacerbates brain swelling (7). Ischaemic oedema is possibly initiated by Na^+^ influx associated with energy failure. Higher osmolarity conditions induce water influx into the cells, resulting in ionic oedema (8). This early oedema phase or cytotoxic oedema may last for several hours prior to the leakage of large volumes of water in the brain, resulting in vasogenic oedema (9). Adverse brain oedema further reduces the blood flow supplying the neurons, causing irreversible apoptosis (10). Studies have shown that the expression of aquaporin-4 (AQP4) is upregulated following cerebral ischaemic injuries (11,12). AQP4 functions in the formation of cerebral oedema and neuronal mortality. Caspase-3 is one of the most critical downstream apoptotic proteases in the caspase cascade ‘waterfall’ and affects neuronal apoptosis (13). Numerous extracellular signals activate caspase-8 and caspase-9 in cerebral ischaemia-reperfusion (I/R) injury. These signals induce caspase-3 to hydrolyse cell-specific proteins and poly (ADP-ribose) polymerase to induce apoptosis (14).

The overexpression of AQP4 and caspase-3 aggravates neural damage following cerebral ischaemic injury. The expression of AQP4 and caspase-3 in brain tissue may be altered by administering an effective drug to relieve cerebral ischaemia. For instance, lactuside B (LB) is a single compound extracted and isolated from the root of *Pterocypsela elata*, which grows in Tongbai County (Henan, China). In our previous study, LB was observed to reduce brain infarct volume and increase the bcl-2/bax mRNA ratio of the cerebral cortex which has an anti-apoptotic effect on nerve cells in rats (15). The hippocampus and the striatum are sensitive areas affected during cerebral ischaemic injury. In the present study, the levels of AQP4 and caspase-3 were evaluated. To study the mechanism by which LB protects against cerebral ischaemia, the effects of LB on AQP4 and caspase-3 mRNA expression in the hippocampus and striatum of rats were investigated.

## Materials and methods

### Animals

Adult male Sprague-Dawley rats (280–320 g; clean grade II) were purchased from the Henan Experimental Animal Centre (Zhengzhou, China; certification no., SYXK Henan 2005–0012). All rats were maintained at a controlled temperature (23±1°C) with a 12-h dark/light cycle and free access to water and food. The rats were cared for in accordance with the guidelines for the treatment of experimental animals published by the Ministry of Science and Technology of the People’s Republic of China in 2006. This study was carried out in strict accordance with the recommendations in the Guide for the Care and Use of Laboratory Animals of the National Institutes of Health (8th version, 2010). The animal use instructions were reviewed and approved by the Institutional Animal Care and Use Committee of Xinxiang Medical University (Xinxiang, China).

### LB

The LB used in this study was provided by the Department of Medicinal Chemistry (Xinxiang Medical University). LB was isolated and purified from 8 kg *P. elata* grown in Tongbai County. LB is a white amorphous water-soluble and chemically stable powder with a purity of >99%.

### Cerebral ischaemia establishment

Focal cerebral ischaemia was induced in the rats by intraluminally occluding the middle cerebral artery (MCA) as described by Longa *et al* (16). The blocking line, a fishing line of size 1.5 with a diameter of 0.2 mm (DaDong Yang, Zhejiang, China), was inserted into the entry point of the MCA from the external carotid arteries (ECAs) via the bifurcation of the common carotid artery and the internal carotid artery (ICA). The line was continuously inserted until the 2.0-cm mark was reached. In the sham surgery group, the lines were inserted into the ICA until the 0.5-cm mark was reached and the remaining surgical procedures were the same as those in the other groups. Following blockage of the arterial flow for 2 h, the lines were withdrawn from the ECAs to allow brain reperfusion. The rats were then returned to their cages and closely monitored. Once the rats had regained consciousness from anaesthesia, they were evaluated for their neurological behavior at various times according to the method described by Longa *et al* (16). The rats with scores from one to four were considered successful models. Neural functional defects were evaluated prior to the rats in each group being sacrificed. A high score indicated the highest severity of neural functional defect.

### Grouping and administration of drugs

A total of 112 rats were randomly divided into five groups: sham surgery; cerebral I/R (I/R); I/R + LB 12.5 mg/kg (I/R + LL); I/R + LB 25 mg/kg (I/R + LM); and I/R + LB 50 mg/kg (I/R + LH). The sham surgery group comprised 16 rats. The remaining four groups comprised 24 rats each. All rats in the sham surgery group survived. In the cerebral ischaemic models, the rat survival rate was 70–80%. Once reperfusion was established, all rats were intraperitoneally injected with 5 ml/kg/day of the corresponding drug. The rats in the sham surgery and model (I/R) groups were also treated with normal saline. The animals in the I/R + LL, I/R + LM and I/R + LH groups were treated with 12.5, 25 and 50 mg/kg LB, respectively. From each group, eight rats were sacrificed 24 h following treatment and used to determine the brain water content. The remaining animals from each group were sacrificed at 24 and 72 h. AQP4 and caspase-3 mRNA expression levels in the hippocampus and the striatum were detected by reverse transcription polymerase chain reaction (RT-PCR).

### Brain water content

Rats were sacrificed at 24 h following focal cerebral ischaemia and their brains were immediately removed. The brain water content was determined as described by Young *et al* (17). A neutral filter paper was used to absorb and remove bloodstains from the brain. The wet weight of each right hemisphere was measured using an FA2004 chemical balance (Shanghai Liangping Instrument Co., Ltd., Shanghai, China) within 90 sec of isolation. Next, the brain was dried in an oven at 110°C for 15 h and the dry weight was obtained. The water content of the brain was calculated using the following equation: Brain water content = (wet weight − dry weight)/wet weight × 100%.

### RT-PCR

RT-PCR was performed to determine the AQP4 and caspase-3 mRNA expression levels in the hippocampus and the striatum. Following sacrifice of the rats, the hippocampus and striatum were obtained and ground in liquid nitrogen. Total RNA was extracted using TRIzol reagent (Invitrogen Life Technologies, Carlsbad, CA, USA) and transcribed to cDNA using a PrimeScript™ RT-PCR kit [Takara Biotechnology (Dalian) Co., Ltd., Dalian, China], in which 1 μl cDNA was used as a template for amplification and 50 μl PCR solution was used. The PCR conditions for AQP4 were set as follows: 95°C for 5 min; 30 cycles of 95°C for 30 sec, 59°C for 30 sec, 72°C for 1 min; and 72°C for 10 min. For caspase-3, the annealing temperature was 54°C and the other conditions were the same as those for AQP4. The PCR products were separated on a 2% agarose gel [Gene tech (Shanghai) Co., Ltd., Shanghai, China] and images of the bands were captured by photography using a Tocan 240 gel imaging system (Tocan Biotechnology Co., Shanghai, China). The greyscale images of each band were detected using Quantity One image analysis software (Bio-Rad, Hercules, CA, USA). The quantities of each PCR product were normalised by dividing the average grey level of the signal by the average grey level of the corresponding β-actin amplicon. These quantities were determined as a semi-quantitative value of the target fragments. The PCR-specific primers of AQP4 (330 bp) and caspase-3 (282 bp), as well as the internal reference primers of β-actin (208 bp), were designed. For AQP4, the upstream and downstream primers were 5′-GGGTTGGACCAATCATAGGCGCT-3′ and 5′-GCAGGAAATCTGAGGCCAGTTCTAGG-3′, respectively. For caspase-3, the upstream and downstream primers were 5′-ACGGTACGCGAAGAAAAGTGAC-3′ and 5′-TCCTGACTTCGTATTTCAGGGC-3′, respectively. For β-actin, the upstream and downstream primers were 5′-CCTTCCTGGGCATGGAGTCCTG-3′ and 5′-GGAGCAATGATCTTGATCTTC-3′, respectively.

### Statistical analysis

Data are presented as mean ± SD. SPSS 17.0 (SPSS, Inc., Chicago, IL, USA) and Excel (Microsoft, Redmond, WA, USA) software were used for statistical analysis. Data were analysed by ANOVA and the Student-Newman-Keuls post hoc test. P<0.05 was considered to indicate a statistically significant difference.

## Results

### Neurological deficit score

Rats in the sham surgery group showed normal activities without any neurological deficit symptoms once consciousness had been regained. Neurological deficits were evaluated and scored at 24 and 72 h following the treatments. Table I shows that the neurological deficit scores were significantly higher in the I/R injury group (24 and 72 h following drug administration, 3.45±0.80 and 3.26±0.62, respectively) than in the sham surgery group (P<0.01). The groups intraperitoneally injected with LB at doses of 12.5, 25 and 50 mg/kg showed decreased neurological deficit scores (24 h following drug administration, 2.60±0.52, 2.23±0.43 and 2.18±0.42, respectively; 72 h following drug administration, 2.45±0.26, 1.88±0.33 and 1.59±0.29, respectively) compared with those in the I/R injury group (P<0.05). The neurological deficit scores of the groups treated with 25 and 50 mg/kg LB at 72 h were significantly lower than those of the groups treated at 24 h (1.88±0.33 and 1.59±0.29; P<0.05). LB decreased the neurological deficit score of the rats following brain I/R injury. This result was evident in the groups treated with 25 and 50 mg/kg LB at 72 h.

### Brain water content

**Figure 1:**
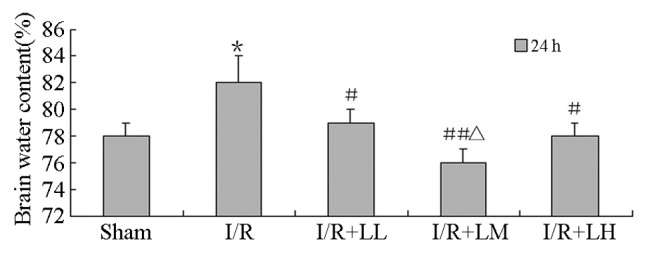
Effect of LB (12.5, 25 and 25 mg/kg) on brain water content following brain I/R injury in rats, all by intraperitoneal injections. Each column represents the mean (%) ± SD. Results were analyzed by ANOVA followed by Student-Newman-Keuls as the post hoc test (each group, n=8). ^*^P<0.05, vs. sham; ^#^P<0.05 and ^##^P<0.01, vs. I/R; ^Δ^P<0.05, vs. I/R + LL and I/R + LH. LB, lactuside B; I/R, ischaemia-reperfusion; LL, 12.5 mg/kg LB; LM, 25 mg/kg LB; LH, 50 mg/kg LB.

Brain water content was determined following drug administration (Fig. 1). Following 2 h MCA occlusion and 24 h reperfusion, the brain water content of the ipsilateral hemisphere in the I/R group significantly increased compared with that in the sham group (82±2 vs. 78±1%; P<0.05). The brain water content significantly decreased in the I/R + LB groups compared with that in the I/R group (LL, 79±1; LM, 76±1; and LH 78±1% vs. 82±2%; P<0.05). LB significantly decreased the brain water content of the rats following brain I/R injury. A dose-response correlation was observed between I/R + LL and I/R + LM groups (76±1 vs. 79±1%; P<0.01).

### RT-PCR

**Figure 2:**
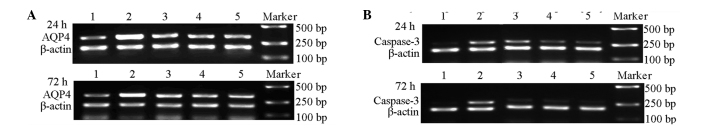
Effect of LB (12.5, 25 and 25 mg/kg) on AQP4 and caspase-3 mRNA expression in the hippocampus. RT-PCR was used to measure (A) AQP4 and (B) caspase-3 mRNA expression levels in the hippocampus. Lane 1, sham; 2, I/R; 3, I/R + LL; 4, I/R + LM; 5, I/R + LH; and Marker. LB, lactuside B; AQP4, aquaporin-4; I/R, ischaemia-reperfusion; LL, 12.5 mg/kg LB; LM, 25 mg/kg LB; LH, 50 mg/kg LB.

Table II and Fig. 2 show that LB decreased AQP4 and caspase-3 mRNA expression in the hippocampus following brain I/R. AQP4 and caspase-3 mRNA expression was significantly higher in the I/R group than in the sham surgery group at 24 and 72 h following drug administration (AQP4, 1.49±0.15 and 1.44±0.13; caspase-3, 0.48±0.05 and 0.50±0.06). AQP4 and caspase-3 mRNA expression was significantly downregulated in the I/R + LB groups (LL, LM and LH groups) at 24 h following drug administration (AQP4, 1.23±0.10, 0.95±0.08 and 0.83±0.07; caspase-3, 0.36±0.06, 0.32±0.05 and 0.29±0.03, respectively) compared with the I/R group. The results observed at 72 h following treatment were similar to those at 24 h (AQP4, 1.24±0.10, 0.86±0.09 and 0.72±0.08; caspase-3, 0.34±0.06, 0.26±0.05 and 0.25±0.04, respectively). The most significant reduction in AQP4 and caspase-3 mRNA expression was noted in the I/R + LM and I/R + LH groups at 72 h following drug administration. This expression showed a dose-dependent correlation.

**Figure 3:**
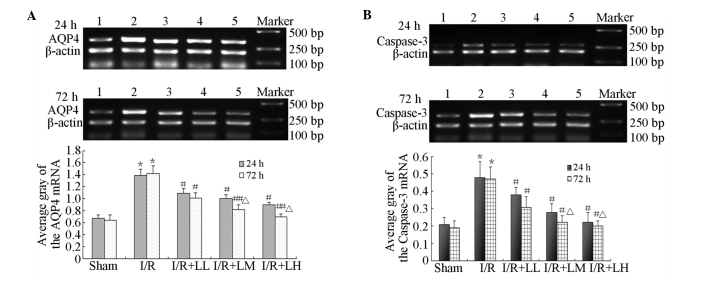
Effect of LB (12.5, 25 and 25 mg/kg) on AQP4 and caspase-3 mRNA expression in the striatum. RT-PCR was used to measure (A) AQP4 and (B) caspase-3 mRNA expression levels in the striatum. ^*^P<0.01, vs. sham group; ^#^P<0.05 and ^##^P<0.01, vs. I/R (model) group; ^Δ^P<0.05, vs. 24 h treatment. Lane 1, sham; 2, I/R; 3, I/R + LL; 4, I/R + LM; 5, I/R + LH; and Marker. LB, lactuside B; AQP4, aquaporin-4; I/R, ischaemia-reperfusion; LL, 12.5 mg/kg LB; LM, 25 mg/kg LB; LH, 50 mg/kg LB.

Fig. 3 shows that LB decreased AQP4 and caspase-3 mRNA expression in the striatum following brain I/R. AQP4 and caspase-3 mRNA expression of the striatum was significantly higher in the I/R group than in the sham surgery group at 24 and 72 h following drug administration (AQP4, 1.39±0.10 and 1.42±0.13; caspase-3, 0.48±0.09 and 0.47±0.07, respectively). LB significantly reduced AQP4 and caspase-3 mRNA expression in the striatum compared with that in the I/R group at 24 h: (AQP4, 1.09±0.08, 1.00±0.06 and 0.90±0.04; caspase-3, 0.38±0.04, 0.28±0.05 and 0.22±0.06, respectively) and 72 h (AQP4, 1.01±0.09, 0.82±0.08 and 0.70±0.05; caspase-3, 0.31±0.06, 0.22±0.04 and 0.20±0.03, respectively) following drug administration. The effects became stronger as the LB doses were increased. The most significant reductions in AQP4 and caspase-3 mRNA expression were noted in the I/R + LM and I/R + LH groups at 72 h following drug administration.

## Discussion

In the present study, AQP4 mRNA was expressed in the hippocampus and the striatum, but the expression levels were low. AQP4 is a protein that functions as a water channel in the central nervous system and is highly expressed in the end-feet of astrocytes. AQP4 allows water to diffuse across the membrane. A normal expression of AQP4 may help maintain water balance in the brain. In this study, AQP4 mRNA expression in the hippocampus and the striatum was significantly higher in the I/R group than in the sham surgery group. This increased expression was considered as mRNA overexpression in these regions. However, specific differences were observed between the peak expression levels of AQP4 mRNA in the hippocampus and striatum. In particular, the peak expression levels in the hippocampus and the striatum were observed at 24 and 72 h following I/R injury, respectively. These results may be induced by the sensitivity of the hippocampus to injury and oedema following cerebral ischaemia. The results indicated that the peak expression of AQP4 mRNA was reached at almost the same time as brain oedema occurred; the peak expression was reached at 1–3 days and then reduced gradually.

As a significant channel of fast-flowing water in acute primary cerebral oedema, AQP4 functions in the exchange of water between normal physiological processes and cerebral ischaemia. Following 2 h MCA occlusion and 24 h reperfusion, serious oedema was observed in the right side of the brain.

The two consistent results show that AQP4 overexpression may elicit a negative effect on cerebral oedema caused by cerebral I/R. Experimental study has also found that LB reduces AQP4 mRNA expression in the hippocampus and striatum in a dose-dependent manner. For instance, Igarashi *et al* (18) observed that an AQP4 inhibitor significantly reduced ischaemic cerebral oedema. Another study noted that AQP4 knockout mice exhibit increased intracranial pressure, in which brain I/R injury is aggravated, showing an enlarged infarct size, a more marked loss of CA1 neurons and astrocyte hypertrophy (19). A constant level of AQP4 expression is capable of attenuating cellular oedema following cerebral ischaemia to a certain degree (20). These studies have hypothesised that the actions of LB against cerebral ischaemia are associated with lower AQP4 mRNA expression levels in the hippocampus and the striatum.

As cerebral ischaemia and cerebral oedema develop, neurons tend to undergo irreversible cell necrosis in the ischaemic core region and may remain viable for several hours or days in the peri-infarct region (penumbra) (14,21). The neurons of the penumbra are prone to apoptosis. Following cerebral ischaemia, the apoptotic cells, which are mainly located in a specific infarcted area, including the preoptic region, corpus striatum, inner margin cortex of infarcted boundary, corpus striatum, hippocampus and olfactory tubercle, reach the highest number in 24–48 h (22). Previous studies have found that caspase-3 expression is increased as cell apoptosis in the brain is increased during local and focal ischaemia (23,24). The present study identified that caspase-3 mRNA expression in the hippocampus and the striatum was significantly higher in the I/R group than in the sham surgery group. However, expression levels were essentially the same at 24 and 72 h following I/R injury. These results are consistent with those observed in a previous study (25). Therefore, the results indicate that LB reduces caspase-3 mRNA expression with a dose-effect correlation in the hippocampus and the striatum. These results also revealed that the actions of LB against cerebral ischaemia are associated with low levels of caspase-3 mRNA expression in the hippocampus and the striatum.

Brain damage caused by cerebral infarction is confined to the cerebral cortex and results in neuronal mortality of the hippocampus and the striatum (26,27). The results of the present study showed that LB reduced the neurological deficit scores and the brain water content. These effects may be associated with the decreased AQP4 and caspase-3 mRNA expression levels in the hippocampus and the striatum following the administration of LB. These results provide novel information concerning the mechanism of action of LB in brain ischaemia. Therefore, LB may potentially be used as a new neuroprotective agent.

## Figures and Tables

**Table I:** Effects of LB on neurological deficits scores following I/R injury in rats (mean ± SD).

Groups	n	Dose, mg/kg	24-h LB treatment	72-h LB treatment
Sham	16	-	0.00±0.00	0.00±0.00
I/R	17	-	3.45±0.80^a^	3.26±0.62^a^
I/R + LL	17	12.5	2.60±0.52^a^,^b^	2.45±0.26^a^,^b^
I/R + LM	18	25.0	2.23±0.43^a^,^c^	1.88±0.33^a^,^c^,^d^
I/R + LH	20	50.0	2.18±0.42^a^,^c^	1.59±0.29^a^,^c^,^d^

a P<0.01, vs. sham group;

b P<0.05 and

c P<0.01, vs. I/R (model) group;

d P<0.05, vs. 24 h treatment.

LB, lactuside B; I/R, ischaemia-reperfusion; LL, LB 12.5 mg/kg; LM, LB 25 mg/kg; LH, LB 50 mg/kg.

**Table II:** Effects of LB on AQP4 and caspase-3 mRNA expression in the hippocampus following I/R injury in rats (mean ± SD).

			24-h LB treatment		72-h LB treatment	
				
Groups	n	Dose, mg/kg	AQP4	Caspase-3	AQP4	Caspase-3
Sham	8	-	0.64±0.06	0.23±0.02	0.64±0.07	0.24±0.04
I/R	9	-	1.49±0.15^a^	0.48±0.05^a^	1.44±0.13^a^	0.50±0.06^a^
I/R+LL	9	12.5	1.23±0.10^a^,^b^	0.36±0.06^a^,^b^	1.24±0.10^a^,^b^	0.34±0.06^a^,^b^
I/R+LM	10	25.0	0.95±0.08^a^,^b^	0.32±0.05^a^,^b^	0.86±0.09^a^,^c^,^e^	0.26±0.05^a^,^b^,^d^
I/R+LH	12	50.0	0.83±0.07^a^,^b^	0.29±0.03^a^,^b^	0.72±0.08^a^,^c^,^e^	0.25±0.04^a^,^b^,^d^

Four rats from each group were sacrificed at 24 h. The remaining rats from each group were sacrificed at 72 h.

a P<0.01, vs. sham group;

b P<0.05 and

c P<0.01, vs. I/R (model) group;

d P<0.05 and

e P<0.01, vs. 24 h treatment.

LB, lactuside B; AQP4, aquaporin-4; I/R, ischaemia-reperfusion; LL, LB 12.5 mg/kg; LM, LB 25 mg/kg; LH, LB 50 mg/kg.
